# Characterization of methylation patterns associated with lifestyle factors and vitamin D supplementation in a healthy elderly cohort from Southwest Sweden

**DOI:** 10.1038/s41598-022-15924-x

**Published:** 2022-07-25

**Authors:** Maria Araceli Diaz Cruz, Benjamin Ulfenborg, Peter Blomstrand, Maria Faresjö, Fredrik Ståhl, Sandra Karlsson

**Affiliations:** 1 Research School of Health and Welfare, School of Health and Welfare, University of Jönköping, Jönköping, Sweden; 2 Department of Biology and Bioinformatics, School of Bioscience, University of Skövde, Skövde, Sweden; 3 Department of Natural Science and Biomedicine, School of Health and Welfare, Jönköping University, Jönköping, Sweden; 4 Department of Biology and Biology Engineering, Chalmers University of Technology, Gothenburg, Sweden; 5 Faculty of Caring Science, Work Life and Social Welfare, Borås University, Borås, Sweden; 6 Department of Clinical Physiology, County Hospital Ryhov, Jönköping, Sweden; 7 Unit of Cardiovascular Sciences, Department of Health, Medicine and Caring Sciences, Linköping University, Linköping, Sweden

**Keywords:** Computational biology and bioinformatics, Genetics, Biomarkers

## Abstract

Numerous studies have shown that lifestyle factors, such as regular physical activity and vitamin D intake, may remarkably improve overall health and mental wellbeing. This is especially important in older adults whose vitamin D deficiency occurs with a high prevalence. This study aimed to examine the influence of lifestyle and vitamin D on global DNA methylation patterns in an elderly cohort in Southwest of Sweden. We also sought to examine the methylation levels of specific genes involved in vitamin D's molecular and metabolic activated pathways. We performed a genome wide methylation analysis, using Illumina Infinium DNA Methylation EPIC 850kBeadChip array, on 277 healthy individuals from Southwest Sweden at the age of 70–95. The study participants also answered queries on lifestyle, vitamin intake, heart medication, and estimated health. Vitamin D intake did not in general affect methylation patterns, which is in concert with other studies. However, when comparing the group of individuals taking vitamin supplements, including vitamin D, with those not taking supplements, a difference in methylation in the solute carrier family 25 (*SCL25A24)* gene was found. This confirms a previous finding, where changes in expression of *SLC25A24* were associated with vitamin D treatment in human monocytes. The combination of vitamin D intake and high physical activity increased methylation of genes linked to regulation of vitamin D receptor pathway, the Wnt pathway and general cancer processes. To our knowledge, this is the first study detecting epigenetic markers associated with the combined effects of vitamin D supplementation and high physical activity. These results deserve to be further investigated in an extended, interventional study cohort, where also the levels of 25(OH)D_3_ can be monitored.

## Background

Individual genetic background and environmental factors are interlaced to lifestyle in determining individual health status. The concept of lifestyle is used to describe the "typical way of life or manner of living characteristic of an individual or group" and has well-documented effects on health, both in terms of disease-free years^1^ and mortality^2^.Although differences in genetic background influence health, gene expression modulated by environmental and lifestyle factors, i.e. through DNA methylation of specific genes^3^, will critically affect the final genetic outcome. Apart from aging, common lifestyle factors known to influence DNA methylation are diet, behaviour, stress, physical activity, psychological stress, smoking, and alcohol consumption[3]and growing evidence suggests that the resulting epigenetic changes may influence a number of age-related disorders^4^.

Vitamin D is a group of fat-soluble secosteroids that act as hormones in the human body and can either be ingested in the diet, taken as supplements, or synthesized in the skin under sunlight exposure^5,6^. Vitamin D has an essential role in human health since its deficiency correlates with various health problems, including osteoporosis^8^, depression^9^, cognitive impairment^10^, cardiovascular disease^11^, hypertension^12^, type 2 diabetes^13^, cancer mortality^14^ and outcome of COVID-19 infection^15^. Moreover, certain groups have a higher prevalence of vitamin D deficiency, such as high pigmented individuals, obese, hospitalized patients, and especially the elderly^16^. In the United States, 35% of adults and 61% of the elderly are vitamin D deficient. In Europe, the percentages are lower in adults (2–30%) but 80% in the institutionalized elderly^8^.The elderly are at risk of lower vitamin D levels due to decreased cutaneous synthesis, bioavailability, and vitamin D intake, as well as other lifestyle changes^16,17^. The metabolism of vitamin D is a multistep process involving several receptors and enzymes. Ergocalciferol (vitamin D_2_) and cholecalciferol (vitamin D_3_) are converted by calciol-25-hydroxylase (CYP2R1) into 25-hydroxycholecalciferol (25(OH)D_3_; calcidiol)^18^. Calcidiol-1α-hydroxylase (CYP27B1 gene) then converts 25(OH)D_3_ into 1,25-dihydroxyvitamin D_3_ (1,25(OH)_2_D)_3_, the biologically active ligand for the vitamin D receptor (VDR). The majority of circulating 25(OH) D_3_ and 1,25(OH)_2_D_3_ in the blood is bound to the vitamin D binding protein (DBP)^19^. The vitamin D-DBP complex is latterly degraded, releasing vitamin D metabolites for physiological action or metabolism^35,36^. A 24-hydroxylase enzyme (*CYP24A1* gene) is in charge of the inactivation of both 25(OH)D_3_ and 1,25(OH)_2_D_3_ via hydroxylation^18^. The active vitamin D metabolite, 1,25(OH)_2_D_3_ or calcitriol, is well known for its effect on gene regulation via its action on the VDR^1^. However, the potential for vitamin D_3_ to regulate gene expression through DNA methylation is currently under investigation^2^. Several epigenetic markers have been shown to be influenced by vitamin D supplementation^20,21^, whereas other studies did not find any significant associations after statistical adjustment or only weak associations^22–25^. So far, the results of the analysis of methylation of vitamin D receptors and associated metabolic enzymes are ambiguous^25–27^.

Physical activity and regular exercise can remarkably improve overall health and mental wellbeing. There is strong evidence that physical activity contributes to increased body function, reduced impairment, independent living, and improved quality of life in the elderly^28^. Regular exercise is also involved in telomere maintenance and the regulation of DNA methylation levels^29,30^. Interestingly, even a single bout of exercise (also termed "acute exercise") has been shown to alter global DNA methylation and specific genes' promoter methylation in skeletal muscle^31^. Combining vitamin D and physical activity has demonstrated synergistic effects in the sarcopenic elderly, such as increasing fat-free mass, strength and functionality, and decreased inflammation^32^. An increase in physical activity has been associated with an increase of circulating 25(OH)D_3_ in men, women, adolescents, and the elderly^33,34^. The mechanisms behind these effects are still unknown, but muscle cells can take up 25(OH)D_3_ from blood and store it by binding with vitamin D binding protein (DBP) during physical activity^19^. Since synergistic effects of vitamin D and physical activity have been demonstrated, this combination may elicit changes in DNA methylation.

Chronological age has been shown to have a profound effect on DNA methylation levels, and several epigenetic markers have been suggested to produce estimations of biological age (referred to as epigenetic age)^36^. However, not much is known about the relationship between epigenetic aging rates and lifestyle factors, such as diet, alcohol consumption and physical activity.

The present study is part of a more extensive cohort study of healthy individuals aged 70–95 in Southwest Sweden, previously described by Gillsjö et al.^37^. The main purpose of this study was to investigate to which extent healthy aging depends on a conscious or unconscious adaption of a lifestyle that matches their genetic predisposition to, for example, different pathological conditions or other health issues. Further, this study aimed to investigate how lifestyle factors affect global methylation patterns and, thus, epigenetic age.

Global methylation patterns associated with levels of vitamin D_3_ combined with other lifestyle habits, such as physical activity, have not been extensively studied in healthy elderly populations. These analyses may be meaningful in the elderly, where vitamin D deficiency and sarcopenia are frequent, and with a higher prevalence of diseases linked to suboptimal vitamin D status. The current study examined the influence of lifestyle factors (physical activity, smoking and alcohol) and vitamin D on global DNA methylation in the healthy elderly cohort mentioned above. Vitamin D intake was estimated through three different sources: Dietary, supplementation and synthesis through sun exposure. Meat and fish intake has been shown to increase the plasma levels of 25(OH)D_3_ whereas vegetarian and vegan diets may result in a lower intake of vitamin D and thus lower plasma concentrations of 25(OH)D_3_^38^. In this study, we investigated dietary intake through fish intake due to its high content in vitamin D_3_. Synthesis of vitamin D_3_ was evaluated from sunlight exposure frequency. The use of sunscreen was also investigated since sun protection may also affect vitamin D_3_ synthesis negatively. Furthermore, we analysed methylation levels of specific genes involved in vitamin D's molecular and metabolic activated pathways.

## Results

The current study investigated the influence of lifestyle factors such as physical activity, smoking, alcohol, and vitamin D_3_ on methylation levels in a healthy elderly cohort from Southwest Sweden. The individuals in this cohort answered a questionnaire, including background data and questions about different lifestyle factors (Additional file 1). Thus, based on the participants' answers to this questionnaire, the cohort was stratified into different study groups (Table S1, Additional file 2). DNA samples from 277 cohort individuals were randomly selected for DNA methylation measurements with microarray experiments. After data processing three samples were removed and 274 samples remained. The background data (general characteristics and medical data) of the cohort participants in the study was pre-processed (three samples were removed) and are presented in Table 1.

**Table 1:** Background data among participants in the methylation study (n = 274).

**General characteristics**			
Gender	Female (n = 188)	Male (n = 81)	Total (n = 269, n/a = 5)
Age (mean ± SD)	75.1 ± 5.8	76.1 ± 4.9	75.4 ± 5.5
**Medical data**			
Self-reported health status (SRHS)			
*Very good*	84	31	115
*Satisfactory*	94	47	141
*Unsatisfactory*	4	4	8
Medicine: Lipids			
*Yes*	130	52	182
*No*	35	20	55
Medicine: Blood pressure			
*Yes*	108	39	147
*No*	70	40	110
Medicine: Heart			
*Yes*	133	51	184
*No*	29	22	51

*n number of samples.

*n/a* no answer.

Differential methylation analyses were performed for all the study groups and for the combination of different lifestyle factors with vitamin D. From these analyses, differentially methylated probes (DMPs) and differentially methylated regions (DMRs) associated with confounding variables and lifestyle factors were identified and further evaluated. Furthermore, the methylation levels of 80 specific genes involved in vitamin D's molecular and metabolic activated pathways (vitamin D-related genes) were assessed. Functional association and associated network analysis provided with an additional overview of the pathways associated with the genes overlapping with the identified DMPs.

### Differentially methylated probes (DMPs) associated with confounding variables: Gender, age, smoking and alcohol habits

**Figure 1:**
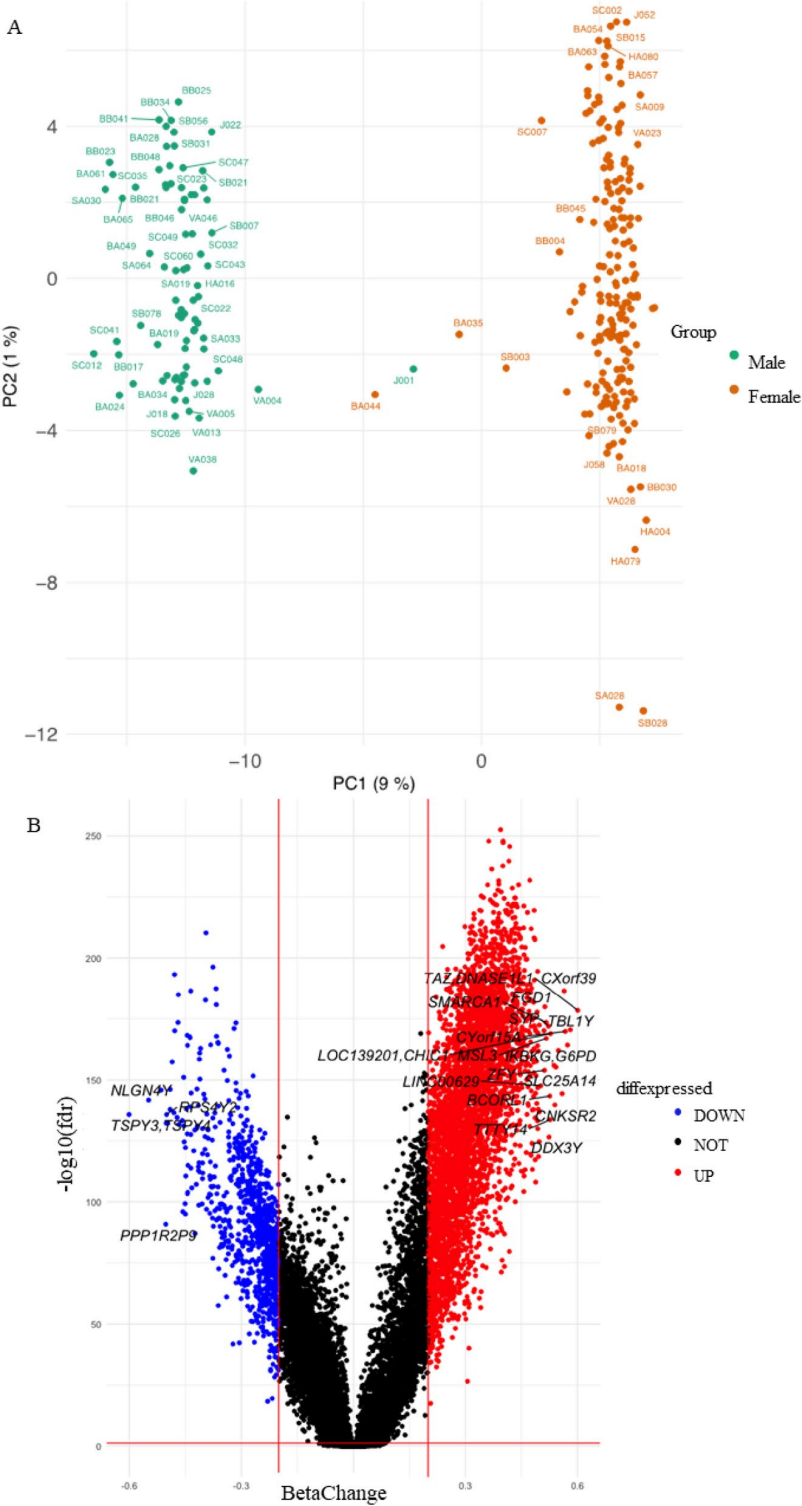
(**A**) PCA on pre-processed methylation data showing differences between female and male individuals in the study (n = 269, n/a = 5). (**B**) Volcano Plot representing DMPs between females (n = 188) and males (n = 81). Red: Hypermethylated probes: differences in beta-values (β) > 0.2 and false discovery rate (FDR) < 0.05. Blue: Hypomethylated probes: β < -0.2 and FDR < 0.05. Black: Non-significant results. Gene names are shown for DMPs with β > 0.5 and FDR < 0.0001, and DMPs with β < -0.5 and FDR < 0.0001.

Principal component analysis (PCA) was performed on the pre-processed methylation dataset to identify potential batch effects in the study (Fig. 1).

The PCA plot shown in Fig. 1A revealed two distinct clusters of samples that corresponded to the gender of participants. 4,329 DMPs and 591 differentially DMRs were identified, separating females from males (Fig. 1B).

Age (Table 1) as well as alcohol habits asstandard glass/week and frequency (Table S1A, Additional file 2) did not show any association with changes in methylation. When analysing smoking habits, 11 DMPs were identified when comparing the group defined as "High" (n = 7) with "None" (n = 149, Table S3, Additional file 3).

### DMPs associated with Vitamin D supplements, fish intake and sun exposure

Participants within the cohort answered whether they were taking vitamin D supplementation or not (Yes/No) and to specify if the vitamin D was part of a multivitamin complex (MC) (Table S1A, Additional file 2). Only one DMP and one DMR were found when comparing the group taking vitamin D as part of a MC (n = 8) with exclusively vitamin D (n = 33), or with not intake of vitamin D, respectively (n = 229, FDR = 0.03, β = 0.2, Table S4, Additional file 3). The DMP was located in the promoter region of the solute carrier family 25 Member 24 (*SLC25A24)* gene in the region from the transcription start site (TSS) to -200 nucleotides upstream (TSS200). No DMPs were found when analysing dietary vitamin D_3_ from fish exclusively (Table S1B, Additional file 2). Vitamin D_3_ synthesis through sunlight exposure or inhibition by the influence of sunscreen (Table S1B, Additional file 2) could not be associated with any methylation changes. The combination of vitamin D supplementation with sun exposure and/or dietary vitamin D_3_ did not result in any significant changes in methylation either.

### DMPs associated with physical activity

Participants within the cohort also answered questions regarding physical activity during winter and summer, respectively. Questions were ranked from 1 to 6 (1 = almost no physical and 6 = hard regularly training several times a week). The physical activity was considered "High" when the sum of the ranks for the summer and winter exercise was ≥ 9 and ≤ 12 (n = 31); “Intermediate,” when it was = 8 (n = 104); and "Low"; when it was < 8 (n = 134). No significant differences in methylation were found from pairwise comparisons between the groups.

Two DMPs in intergenic regions were found when comparing individuals reporting moderate to high physical activity (n = 262) with very low or to almost no physical activity (n = 7, Table S5, Additional file 3).

### DMPs associated with Vitamin D supplements and high physical activity

A total of 357 DMPs, corresponding to 221 genes and 108 intergenic regions, and 9 DMRs, were identified when comparing the group taking vitamin D supplements and exerting high level of physical activity (n = 7) with the group of individuals not taking vitamin D supplements and exerting low levels of physical activity (n = 117, Tables S6–S7, Additional file 3). Table 2 shows the 20 DMPs with the highest statistical significance. Totally 74 out of the 357 DMPs (21%) were located in promoter regions (Table S8, Additional file 3). No differences in methylation were found between the group taking vitamin D supplementation and intermediate physical activity (n = 17) compared with not taking vitamin D supplements and exerting only low level of physical activity (n = 117).

**Table 2:** DMPs in the group of individuals taking vitamin D supplements and exerting high levels of physical activity.

Gene name	Gene symbol	Probe	Chr	FDR	β
*Dab, mitogen-responsive phosphoprotein, homolog 2*	*DAB2*	cg21886364	5	6.5E-07	0.28
*Collagen, type XXIII, alpha 1*	*COL23A1*	cg07145979	5	7E-07	0.28
*Superoxide dismutase 3, extracellular*	*SOD3*	cg17573292	4	2.7E-06	0.34
*SKI proto-oncogene*	*SKI*	cg13488570	1	4.5E-06	0.23
Lipin 1	*LPIN1*	cg00523161	2	4.5E-06	0.34
	Intergenic region	cg24792289	5	4.5E-06	0.27
	Intergenic region	cg23244910	6	6.9E-06	0.28
GLI family zinc finger 3	*GLI3*	cg06310816	7	2.1E-05	0.20
*LOC100132111, C2 calcium-dependent domain containing 4D*	*C2CD4D*	cg26426745	1	2.4E-05	0.35
*Rabphilin 3A-like*	*RPH3AL*	cg15295273	17	2.4E-05	0.31
*Reticulon 4 receptor-like 1*	*RTN4RL1*	cg08454053	17	3.2E-05	0.24
KIAA0319-like	*KIAA0319L*	cg22698544	1	7.1E-05	0.26
	Intergenic region	cg05964935	Y	7.1E-05	-0.29
*NK2 homeobox 8*	*NKX2-8*	cg20008148	14	8.8E-05	0.36
*Neurotrimin*	*NTM*	cg04934246	11	2.3E-04	0.21
	Intergenic region	cg16668359	2	2.3E-04	0.31
*Nuclear factor I/X*	*NFIX*	cg20116828	19	3E-04	0.38
	Intergenic region	cg20586840	4	3.3E-04	-0.21
*IQ motif and Sec7 domain 1*	*IQSEC1*	cg16562217	3	3.7E-04	0.56
	Intergenic region	cg13100965	7	3.8E-04	0.31

*FDR False discovery rate; *Chr* chromosome, *β* beta value difference.

### Methylated genes induced by 1,25(OH)_2_D_3_ and high physical activity are associated with global regulation of transcription

Functional annotation analysis was performed with PANTHER for all 221 genes containing DMPs for the vitamin D_3_ and high physical activity group. Table 3 shows the most enriched terms (False discovery rate (FDR) < 0.05) for biological process (BP) and molecular function (MF).

**Table 3:** Functional annotation analysis of differentially methylated genes associated with vitamin D supplements and high physical activity group.

GO category	Term	# genes	FDR
Molecular function (MF)	RNA polymerase II cis-regulatory region sequence-specific DNA binding	30	0.02
	DNA-binding transcription activator activity, RNA polymerase II-specific	17	0.03
	Protein binding	181	0.04
Biological process (BP)	Positive regulation of transcription by RNA polymerase II promoter	35	0.002
	Cell adhesion	27	0.007
	Regulation of cell population proliferation	36	0.03

*^#^ genes: Number of genes, *FDR* False discovery rate.

The enriched terms reported by PANTHER (BP and MF) were related to processes and mechanisms related to transcriptional regulation (Table 3). The most significant MF and BP were related to a regulation of the transcription in the cis-regulatory element or promoter of the RNA polymerase II. There were 22 genes overlapping between MF and BP: hematopoietically expressed homeobox (*HHEX*), distal-less homeobox 1 (DLX1), nuclear factor I/X (*NFIX*), zinc finger protein 721 (*ZNF721*), pleiomorphic adenoma gene-like 1 (*PLAGL1*), transcription factor AP-2 epsilon (*TFAP2E*), T-box 1 (*TBX1*), SKI proto-oncogene (*SKI*), homeobox D13 (*HOXD13*), FBJ murine osteosarcoma viral oncogene homolog B (*FOSB*), runt-related transcription factor 1 (*RUNX1*), distal-less homeobox 5 (*DLX5*), forkhead box I1 (*FOXI1*), GLI family zinc finger 3 (*GLI3*), zinc finger protein 639 (*ZNF639*), transcription factor CP2 (*TFCP2*), orthodenticle homeobox 1 (*OTX1*), NK2 homeobox 8 (*NKX2*-8), regulatory factor X, 5 (*RFX5*), nuclear receptor subfamily 2, group F, member 1 (*NR2F1*), nuclear factor of activated T-cells, cytoplasmic, calcineurin-dependent 4 (*NFATC4*), and Spi-1 proto-oncogene (*SPI1*).

Network analysis with GeneMania showed that genes related to hypomethylated probes and their associated genes were involved in "Sodium ion transport" processes (Figure S1, Additional file 4, FDR = 0.005 – 0.03).

### Methylated genes induced by 1,25(OH)_2_D_3_ and high physical activity are linked to the regulation of the Vitamin D receptor, Wnt signaling, and cancer pathways

**Figure 2:**
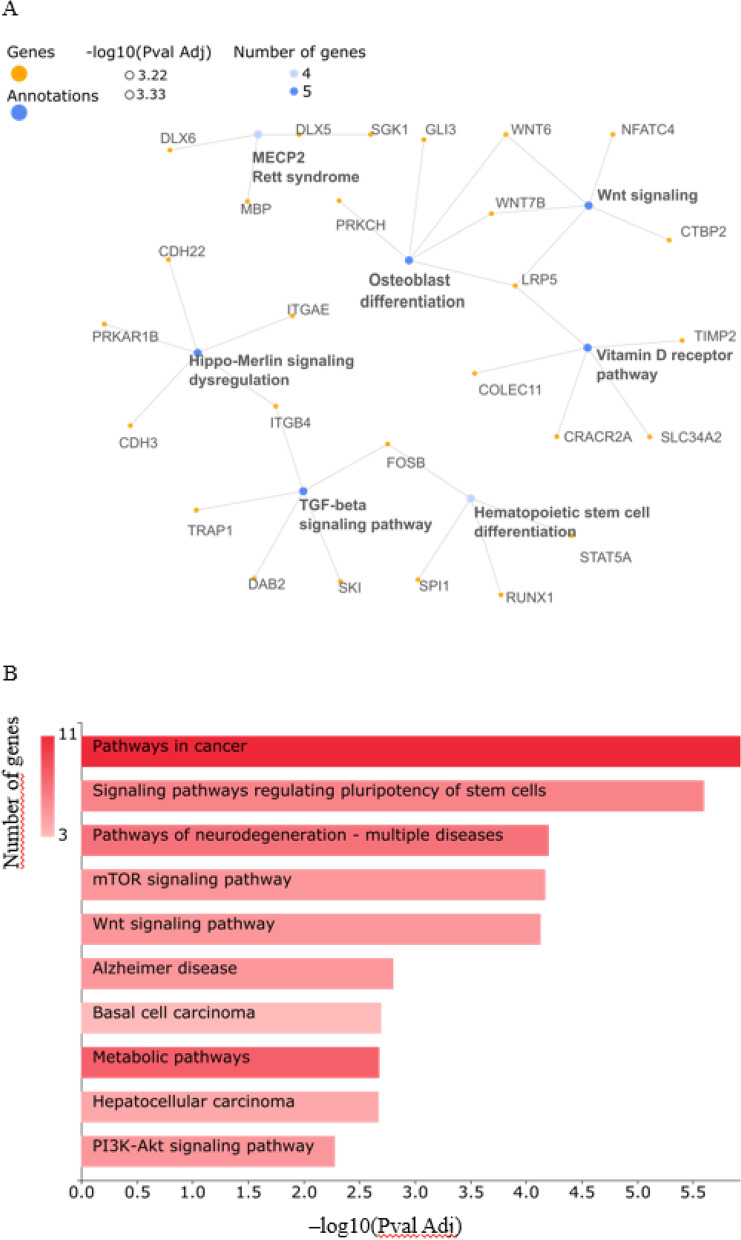
Main regulated pathways related to genes with differentially hypermethylated probes found in the vitamin D and high physical activity group. (**A**) Top 7 significant regulated pathways obtained with Wikipathways in GeneCodis, adjusted *P* value < 0.05. (**B**) Top 10 significant regulated pathways obtained with KEGG, adjusted *P* value < 0.05.

Genes corresponding with differentially hypermethylated and hypomethylated probes, for the vitamin D_3_ and high physical activity group were separately input into GeneCodis to analyse the main regulated pathways. Figure 2 shows the main regulated pathways for the genes corresponding with differentially hypermethylated positions. The three most significant pathways obtained from WikiPathways analyses were: Vitamin D receptor, transforming growth factor beta (TGF-β), and the Wnt signaling pathway (Fig. 2A). The three most significant cellular processes/pathways extracted from KEGG were cancer, stem cell pluripotency, and neurodegeneration in multiple diseases (Fig. 2B).

### Methylation status of vitamin D-related genes

**Figure 3:**
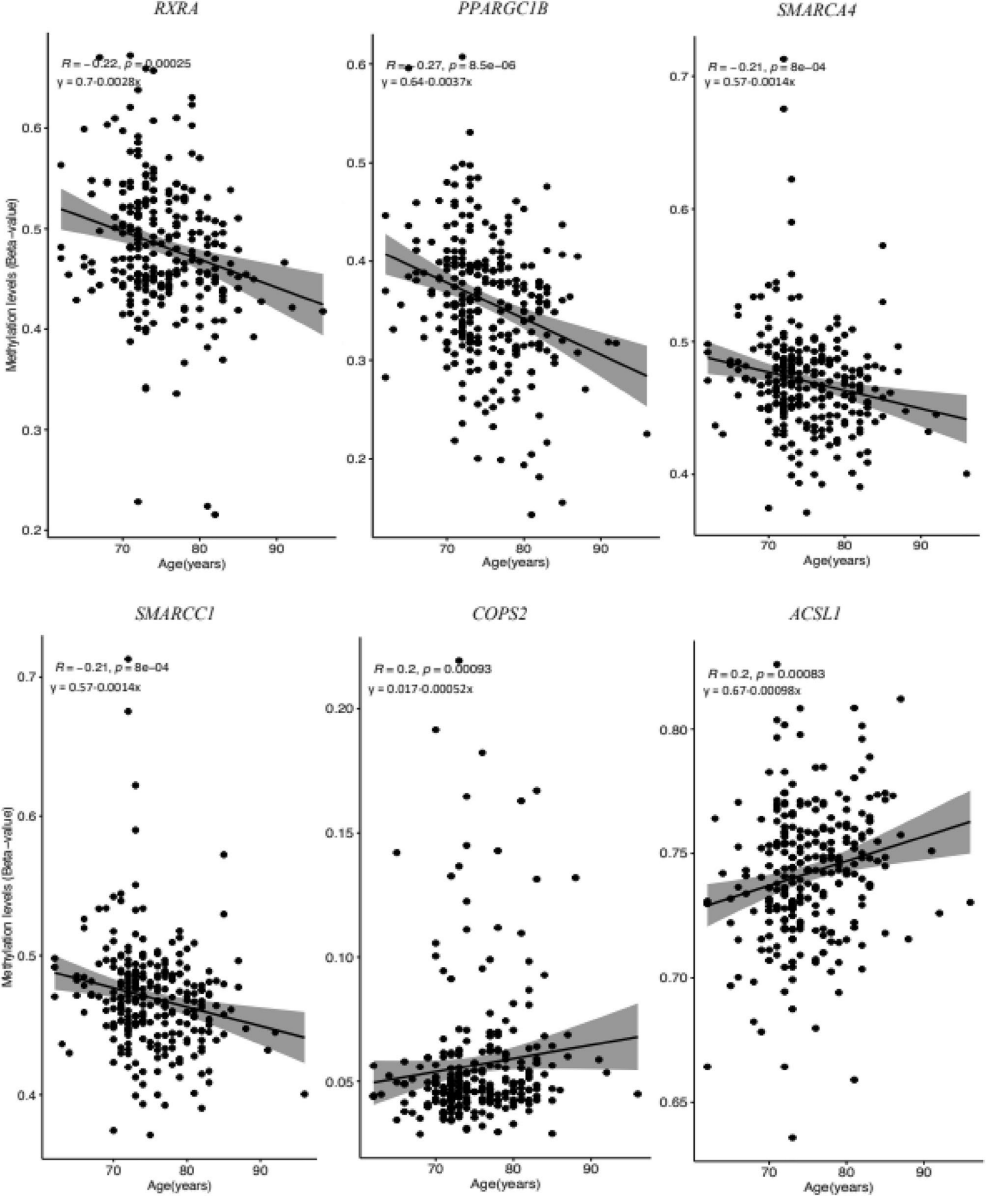
Association of methylation levels of CpGs at *RXRA*, *PPARGC1B*, *SMARCA4*, *SMARCC1*, *COSP2,* and *ACSL1*; and age of the individuals in the study. Association was evaluated by Spearman's correlation method (r2, *P* value < 0.05).

Methylation levels of the 80 vitamin D-related genes, including vitamin D receptors and metabolic enzymes among others (Table S2, Additional file 2) showed weak correlations with different factors analysed in the study. The methylation levels of 12 CpG sites in 8 of the 80 vitamin D-related genes, showed a correlation value r2 ≈ 0.2, (Table S9, Additional file 4). Age showed a negative association with the methylation levels of *retinoid X receptor, alpha* (*RXRA)* (r2 = -0.22, adjusted *P* value = 0.001), *peroxisome proliferator-activated receptor gamma, coactivator 1 beta (PPARGC1B)* (r2 = -0.27, adjusted *P* value = 0.000009), *SWI/SNF related, matrix associated, actin dependent regulator of chromatin, subfamily c, member* 4 ***(****SMARCA4* (r2 = -0.21, adjusted* P* value = 0.00080) and SWI/SNF related, matrix associated, actin dependent regulator of chromatin, subfamily c, member 1 (*SMARCC1)* (r2 = -0.21, adjusted *P* value = 0.00080) (Fig. 3). On the contrary, age showed a positive association with methylation levels of *COPS2* (r2 = -0.20, adjusted *P* value = 0,00,093) and *acyl-CoA synthetase long-chain family member 1 (ACSL1)* (r2 = -0.20, adjusted *P* value = 0.00083) (Table S9, Additional file 4 & Fig. 3).

Methylation status of the 80 vitamin D-related genes did not change with vitamin D supplementation (FDR = 0.5–0.9). There were no significant changes in methylation associated with the rest of the binary factors, including vitamin supplements, medicines (lipids, heart, and blood pressure), self-reported health status (SRHS) and alcohol (frequency/week).

## Discussion

Global methylation patterns associated with intake of vitamin D supplements combined with other lifestyle habits, such as physical activity, have not been thoroughly studied in elderly populations. Herein, we assessed the influence of lifestyle habits, including vitamin D intake, on global DNA methylation patterns in an elderly cohort in Southwest of Sweden. Further, we examined the methylation status of vitamin D-specific genes involved in vitamin D's molecular and metabolic activated pathways.

In the present study, vitamin D intake from diet and supplementation and sunlight exposure or the use of sunscreen did not affect, in general, the methylation patterns in the elderly. Effects in methylation could only be observed when comparing groups with individuals taking vitamin supplements, including vitamin D, with individuals not taking vitamin supplements. Thus, there was an increase in the methylation within the promoter region of the *SLC25A24 g*ene. *SCLC25A24* encodes for the calcium-binding mitochondrial carrier protein (*SCaMC-1*), one of five ATP-Mg/P_i_ carriers with the vital function to exchange ATP-Mg or ADP for phosphate across the mitochondrial inner membrane. It has been shown that *SLC25A24* mutations lead to impaired mitochondrial ATP synthesis and cause hyperpolarization and increased proton leakage, which results in decreasing energy metabolism^39^. In addition, genetic analyses in humans and mouse models have suggested that SLC25A24 acts as an essential regulator of body fat mass and adipogenesis^40^. In line with our results, *SLC25A24* has been reported as a primary vitamin D-activated target. Changes in the expression levels in the TSS of the gene were observed within four hours of stimulation with 1,25(OH)_2_D_3_ in human monocytes^41^. The previous study and our results indicate a role of vitamin D in regulating transcriptional levels of *SLC25A24*.

Only a few studies have examined the relationship between vitamin D supplementation and methylation patterns. Nair-Shalliker et al*.* showed that exposure to sunlight was inversely associated with global DNA methylation and that vitamin D levels did not influence this association^24^. Chavez Valencia et al*.* exposed primary human blood mononuclear cells to calcitriol for up to 120 h and showed that DNA methylation patterns did not change in response to vitamin D treatment^23^. Tapp et al*.* investigated the effects of several nutritional factors and age-related DNA methylation in the human rectal mucosa and reported a weak positive correlation between vitamin D and *LINE-1* methylation^20^.

We have also evaluated the influence of other lifestyle habits on methylation levels, such as general vitamin intake, eating habits (fish intake), and physical activity. In general, the global methylation patterns did not change according to the lifestyle factors evaluated in this study. However, two DMPs in intergenic regions were found when comparing individuals who exerted moderate-high physical activity with those who exerted very low or almost no physical activity. Several studies have shown that both lifelong (chronic) and acute physical activity affect DNA methylation in the skeletal muscle, blood, and saliva, and several epigenetic markers have been suggested^29,42–44^. However, these studies reported epigenetic modifications as outcomes of exercise intervention. Our study is a cross-sectional study, where hours per week of physical activity and relative intensities of the exercise were self-reported in a questionnaire. Thus, we recognize that the study participants' estimations of the intensity of the physical activity might affect this type of retrospective study.

In a previous study, Rondanelli et al*.* could show that combining vitamin D intake and physical activity resulted in synergistic effects in counteracting sarcopenia in the elderly, such as increasing muscle and strength^32^. Moreover, physical activity has been associated with an increase of circulating 25(OH)D_3_^45,46^ which may be due to active cycling of 25(OH)D_3_ between skeletal muscle and blood^47^. Moreover, vitamin D supplementation in combination with resistance exercise in elderly, results in increased muscle mass^48^ and has been discussed as a useful strategy against sarcopenia^49^.. The isolated effects of vitamin D supplementation and physical activity on methylation have been evaluated previously, but not their combined effects. High levels of physical activity together with vitamin D supplementation were associated with DMPs in 221 genes and 108 intergenic regions. Out of these DMPs, 21% were located within promoter regions suggesting a role in transcriptional regulation. Functional analyses, of the genes corresponding to all the DMPs, showed that a great proportion of the genes were related to the regulation of the transcription by RNA polymerase II.

Interestingly, some genes containing the hypermethylated DMPs were involved in regulating the vitamin D receptor pathway. Thus, we speculate that high physical activity may increase vitamin D bioavailability by increasing the binding of 25(OH)D_3_ with DBP and, in the end, regulating the transcription of genes related to the vitamin D receptor. Other genes containing the hypermethylated DMPs were involved in Wnt-signaling and pathways implicated in cancer development. The increase of methylation levels in these genes point to an inhibition of the transcriptional activity in cancer and Wnt-signaling pathways. These results are in line with previous studies, where 1,25(OH)_2_D_3_ and physical activity inhibited activation of the Wnt/β-catenin signalling, a pathway frequently hyperactive in cancer^50,51^.

We also analysed the methylation status of 2,113 CpG sites in 80 vitamin D-related genes, including well-known receptors, metabolic enzymes, and primary activated targets of vitamin D, identified from the literature. Thus, we found an inverse correlation between *RXRA* methylation and age. Intriguingly, a previous study using computational networks identified that one of the most critical key nodes related to chronological age across multiple tissue types was *RXRA* methylation^52^.

The methylation levels of the vitamin D-related genes examined here did not change in individuals with a vitamin D-containing diet, vitamin D supplementation, or sun exposure. Results from previous studies of methylation of vitamin D receptors and associated metabolic enzymes in response to vitamin D supplementation are inconsistent and contradictory^25–27^. Zhou et al*.* reported that the methylation status of cytochrome P450 family 2 subfamily R member 1 **(***CYP2R1)* and cytochrome P450 family 24 subfamily A member 1 (*CYP24A1)* were negatively associated with 25(OH)D baseline plasma levels^27^. However, these associations were weak and disappeared when the prediction was corrected for vitamin D intake^27^. On the other hand, Beckett et al*.* showed that calcium intake, age, sex, BMI, cigarette smoking history, alcohol intake, and cumulative irradiance increased the association with 25(OH)D_3_ plasma levels^26^. However, this study was only conducted on peripheral blood cells, and the cohort was of limited size^26^.

Several other factors could have influenced the results of our analyses, for example the choice of saliva as microfluid for epigenomic profiling, gender, age and lifestyle factors such as smoking, and alcohol consumption. In this study, saliva was chosen, instead of peripheral blood mononuclear cells (PBMC), due to several reasons. Mainly since the study participants were recruited from the organisation Active seniors at seminars held outside the health care. Thus, choosing saliva made the sample collection easier and less invasive. The sample handling is much more simplified as there was no need for the addition of an anticoagulant to avoid clotting upon collection and the risk of disease transmission that may occur when in contact or via needle prick is lower than with blood sampling. Only a few studies have compared DNA methylation patterns in blood and saliva^53–56^. However, when comparing global methylation patterns, Smith et al.^57^ observed the saliva methylome to be positively correlated with methylation in blood for 88.5% of the CpG sites analysed on the Illumina 450 K arrays. Other studies indicate that the majority of CpG sites are similarly methylated in blood and saliva with 1.8–4%.of the CpG islands differentially expressed between PBMC and saliva^53,55^.

When analysing differences in methylation depending on gender, a total of 4,329 DMPs and 591 DMRs were identified. This is reflected in the PCA showing a clear separation between male and female epigenetic signatures and is in agreement with other studies of sex-differences in the epigenetics literature^58,59^. As an exploratory technique, PCA highlights the major sources of variation in the data and will therefore capture sex-differences as these explain a large fraction of total variance. Less pronounced effects that explain a smaller fraction of total variance, such as vitamin D intake-related changes, are therefore masked when looking at the genome-wide epigenetic signal. In the case of smoking, a few DMPs were found when the group of higher cigarette consumption was compared with the group that did not smoke. Several studies have demonstrated an association between age and alcohol consumption^60–63^, but in this cohort, no associations were found when comparing age and alcohol consumption. This could be due to a narrow age range within the cohort, and that the group that reported high consumption of alcohol (standard glass/week) was reduced in size, respectively.

In summary, we have examined the influence of lifestyle and vitamin D intake on global DNA methylation patterns in an elderly cohort in Southwest of Sweden. An association between age and the methylation of *RXRA* was found, which confirms previous observations of epigenetic clocks. We could also show that the combination of vitamin D supplementation and a high level of physical activity elicited changes in DNA methylation in genes regulating the vitamin D receptor pathway, implying that physical activity and vitamin D supplements may lead to transcription of genes related to the vitamin D receptor and pathways. However, since the group containing individuals taking vitamin D supplements and exerting high levels of physical activity was relatively small compared to the group of individuals not taking vitamin D supplements and exerting low levels of physical activity, these results need to be verified in a comprehensive interventional study. Also, since the samples for DNA extraction were collected from the saliva, local effects on DNA methylation in the skeletal muscle tissue cannot be detected. On the other hand, our results demonstrate global methylation effects from physical activity and vitamin D supplementation.

## Conclusions

In this study, we show that lifestyle factors, i.e., moderate to high levels of physical activity combined with the intake of vitamin D supplements, affect DNA methylation in genes regulating the vitamin-D-receptor pathway and pathways associated with cancer initiation and development. Vitamin D deficiency is abundant among the elderly and considering different choices in lifestyle may substantially contribute to healthy aging. To our knowledge, this is the first study showing epigenetic changes associated with the combined effects of vitamin D supplementation and high physical activity in an elderly population.

## Methods

### Study participants

The elderly cohort analysed in this study included community-dwelling individuals recruited in collaboration with the association Active Seniors (Aktiva seniorer)—a nationwide independent political association in Sweden for older adults. A total of 800 individuals in Southwest Sweden, at age 70 to 95, were invited to participate in the study, as described in^37^. At the seminars, the participants were informed about the study and asked to participate. Altogether, 530 participants answered a questionnaire including background data and different lifestyle habits (Additional file 1). Out of the 530 participants, 10 individuals choose to participate solely in the questionnaire study. Thus, saliva samples for subsequent DNA extraction were collected from 520 individuals and kept in a freezer at -20 °C. Of the 520 saliva samples, 277 samples were randomly selected for methylation analyses. After data processing three samples were removed and 274 samples remained.

### Study groups

The cohort was stratified into study groups based on participants' answers to general characteristics (age and gender), medical data (Table 1) and questions about different lifestyle factors, including vitamin supplementations, smoking and alcohol habits, physical activity and sunbathing habits. (Table S1, Additional file 2). Vitamin D intake was evaluated from the vitamin D supplementation and dietary vitamin D (Fish and seafood frequency, Table S1, Additional file 2). To estimate potential vitamin D synthesis in the skin, sunlight exposure and use of sunscreen were analysed (Sun exposure and use of sunscreen, Table S1B, Additional file 2). Physical activity during the whole year was evaluated from the combination of answers to physical activity in summer and in winter (Physical activity during summer and winter, Table S1A).

### DNA methylation analysis

DNA was extracted from 1 mL of saliva using SalivaGene Collector and PSP SalivaGene DNA Kit (Stratec, Berlin, Germany). DNA quality (Abs_260_/Abs_280_ ratio), purity (Abs_260_/Abs_230_ ratio), and concentration were assessed spectrophotometrically using Nanodrop. A total of 500 ng of extracted DNA with a quality ratio > 1.8 and with a purity ratio ~ 2.0 was submitted for genome-wide array analysis to Swegene Centre for Integrative Biology at Lund University (SCIBLU), Lund, Sweden. Global methylation patterns were measured using the Illumina Infinium DNA Methylation EPIC 850 k BeadChip array (Illumina, San Diego, CA, USA) to interrogate methylation in 850,000 probes or 285,000 genes/markers, respectively, across the genome.

The raw intensity files (idat) were exported from Genome Studio software (Illumina, San Diego, CA, USA) and imported into R version 4.1 (R Core Team 2021) for further analyses. Annotation, pre-processing, quality control (QC), and differential methylation analyses were performed with Miodin (MultI-Omics Data INtegration) package v.0.5.4^64^. Probes were annotated with the “Infinium MethylationEPIC v1.0 B4” manifest file available at the Illumina website (https://support.illumina.com/downloads.html). Data was pre-processed by first removing non-CpG probes and those close to SNPs[65]or found to be cross-hybridizing^66^. Data were also filtered on coefficient of variation (CV) to remove probes with CV < 0.1, followed by Beta-Mixture Quantile (BMIQ) normalization^67^ and cell composition correction^68^. After pre-processing, a total of 198,550 probes were filtered out and 667,259 remained. Potential variation or batch effects on the methylation data were assessed by principal component analysis (PCA). Three samples were considered outliers and removed after PCA, and 274 remained for the downstream analysis.

### Differential methylation analysis

Differential methylation analysis was performed for all the study groups associated with background data (Table 1) and lifestyle habits (Table S1, Additional file 2). Moreover, these analyses were done for the combination of different lifestyle factors with vitamin D (dietary, supplementation, and synthesis). The differential methylation analyses were conducted in Miodin v0.6.0 with the DMRcate package version 2.2.3^69^. Gender, age, smoking and alcohol (standard glass/week and frequency), were included as covariates in the analyses. *P* values were adjusted for multiple testing with Benjamini–Hochberg correction. Methylated probes or CpG sites, and regions, with a difference in mean beta value (β) between groups of β ≥ 0.2 or ≤ -0.2 and false discovery rate (FDR) < 0.05, were considered differentially methylated. DMPs were considered "hypomethylated" if β ≤ -0.2, and "hypermethylated" if β ≥ 0.2. Genes overlapping with the DMPs were used as input for the functional annotation and network analyses.

### Functional annotation and associated network analysis

Functional annotation analysis was performed using the GO enrichment analysis with PANTHER 16.0^70,71^. The main regulated pathways were extracted from the pathway databases KEGG (Kyoto Encyclopedia of Genes and Genomes) and WikiPathways in GeneCodis 4.0^72^. Genes overlapping with differentially hypomethylated and hypermethylated probes were input into the different tools separately and together. Network analysis was performed using GeneMANIA prediction web server (University of Toronto, www.genemania.org^73^. GeneMANIA was used to detect related genes, potential interactions, and associated networks to the input genes from datasets with available genomics and proteomics data. Maximal resultant genes were set to 30 and maximal resultant attributes to 20. Functionally enriched pathways and results from the associated network analysis were considered significant if FDR < 0.05.

### Analysis of methylation status of vitamin D-related genes

To examine the association between methylation levels in vitamin-D-related genes with each study group, a total of 80 vitamin D-related genes corresponding to 2,113 CpG sites were selected for further analyses. The selected genes included the classical vitamin D receptor (*VDR)* and the putative vitamin D receptor protein disulfide isomerase 3 (*PDIA3*, metabolic enzymes (*CYP27A1*, *CYP27B1*, *CYP2R1,* and *CYP24A1*), secondary activated receptors (*RXRA* and *TRPV6*), vitamin D binding protein (*DBP*), and primary activated targets of vitamin D, previously reported in literature and databases (Table S2, Additional file 2). Statistical analyses on the selected genes were performed in R. Analysis of the association of methylation levels of vitamin D genes with non-binary factors was carried out using Spearman's correlation test with the Hmisc packaged v4.6.0. Differences in methylation levels for binary factors were assessed with Welch's two samples t-test considering unequal variances. Methylation values were corrected using an empirical Bayes framework with parametric adjustment^74^ for gender with the sva package v.3.36.0. *P* values were adjusted for multiple testing with Benjamini–Hochberg correction.

### Ethics approval and consent to participate

This study followed the Declaration of Helsinki^75^ and was approved by the Regional Ethical Review Board in Gothenburg (Dnr: 989–13). Written informed consent was obtained from all participants.

## Supplementary Information

Supplementary Information 1.

Supplementary Information 2.

Supplementary Information 3.

Supplementary Information 4.

## Data Availability

The DNA methylation data have been deposited in ArrayExpress database at EMBL-EBI [www.ebi.ac.uk/arrayexpress] under Accession Number E-MTAB-11445.

## Abbreviations

CYP2R1: Calciol-25-hydroxylase
CYP27B1: Calcidiol-1α-hydroxylase
CYP24A1: 24-Hydroxylase
DBP: Vitamin D binding protein
DMP: Differentially methylated probe
DMR: Differentially methylated region
FDR: False discovery rate
PCA: Principal component analysis
PDIA3: Protein disulfide-isomerase A3
SHRS: Self-reported health status
TGF-β: Transforming growth factor beta
TSS: Transcription start site
VDR: Vitamin D receptor
