# Supplementary material for: Characterization of methylation patterns associated with lifestyle factors and vitamin D supplementation in a healthy elderly cohort from Southwest Sweden
Source: Sci Rep. 2022 Jul 25;12:12670. doi: 10.1038/s41598-022-15924-x (PMC9310683; doi:10.1038/s41598-022-15924-x)
Supplement: Supplementary file 1 — Supplementary Information 1. [file 41598_2022_15924_MOESM1_ESM.docx]

**Questionnaire**: *To live as a pensioner, A questionnaire about the importance of lifestyle for health*

**Background Data**

Year of birth:

Female **☐** Male **☐**

I estimate my general health condition as:

Very Good **☐** Good **☐** Not Good**☐**

Are you taking medicine for:

Blood Pressure: Yes **☐** No **☐**

Blood Fat: Yes **☐** No **☐**

Heart: Yes **☐** No **☐**

**Vitamin supplements**

**Are you taking any vitamin or food supplements?** (open answer)

**Smoking habits**

*Adapted from the Public Health Agency of Sweden – Questionnaires*

**What do your smoking habits look like?**

Level 1: I have never been a smoker.

Level 2: I quit smoking more than 16 years ago.

Level 3: I quit smoking between 10 and 16 years ago.

Level 4: I quit smoking between 3 and 9 years ago.

Level 5: I quit smoking less than 3 years ago.

Level 6: I smoke, but not daily.

Level 7: I smoke 1-9 cigarettes daily.

Level 8: I smoke 10-19 cigarettes daily.

Level 9: I smoke 20 cigarettes or more a day.

**Alcohol habits**

*Adapted from the Public Health Agency of Sweden – Questionnaires*

**How many standard glasses do you drink in a normal week?**

Level 1: Less than 1 standard glass per week, or not at all.

Level 2: 1-4 standard glasses per week.

Level 3: 5-9 standard glasses per week.

Level 4: 10-14 standard glasses per week.

Level 5: 15 or more standard glasses per week.

**How often do you drink a standard glass or more during a regular week?**

Level 1: 0-2 days a week.

Level 2: 3-7 days a week.

**Physical Activity**

*Adapted from Grimby G. Physical activity and muscle training in the elderly. Acta Med Scand 1986;711:233‐237*

**How physically active do you estimate that you have been during the last summer semester?**

Level 1. Hardly any physical activity

Level 2. Mostly sitting, sometimes a walk, light gardening, or similar tasks

Level 3. Light physical exercise around 2-4 h a week, such as walks, fishing, dancing, ordinary gardening including walks to and from shops.

Level 4. Moderate exercise 1-2 h a week, such as jogging, swimming, gymnastics, heavy gardening, home-repair, or light physical activities more than 4 h a week.

Level 5. Moderate exercise as least 3 h a week, such as tennis, swimming, jogging

Level 6. Hard or very hard exercise regularly and several times a week, where the physical exertion is great, such as jogging

**How physically active do you estimate that you have been during the last winter semester?**

Level 1. Hardly any physical activity

Level 2. Mostly sitting, sometimes a walk, light gardening, or similar tasks

Level 3. Light physical exercise around 2-4 h a week, such as walks, fishing, dancing, ordinary gardening including walks to and from shops.

Level 4. Moderate exercise 1-2 h a week, such as jogging, swimming, gymnastics, heavy gardening, home-repair, or light physical activities more than 4 h a week.

Level 5. Moderate exercise as least 3 h a week, such as tennis, swimming, jogging

Level 6. Hard or very hard exercise regularly and several times a week, where the physical exertion is great, such as jogging

**Sunbathing habits**

**To what extent do you sunbathe when given the opportunity (during the summer or when staying at southern latitudes)?**

Levels 1 – 5

1: Almost not at all

5: Very much

**Do you use sunscreen when sunbathing?**

Levels 1-3

Levels 1-3

1: Never

3: Always

**Eating habits**

*Adapted from the Swedish National Board of Health and Welfare, Simple advice for good eating habits (articleno: 2014-6-24)*

**How often do you eat fish or seafood as a main course, in a salad or as a side dish?**

Level 1: Twice a day or more often.

Level 2: Once a day.

Level 3: A few times a week.

Level 4: Once a week or less frequently.
