# Supplementary material for: Characterization of methylation patterns associated with lifestyle factors and vitamin D supplementation in a healthy elderly cohort from Southwest Sweden
Source: Sci Rep. 2022 Jul 25;12:12670. doi: 10.1038/s41598-022-15924-x (PMC9310683; doi:10.1038/s41598-022-15924-x)
Supplement: Supplementary file 2 — Supplementary Information 2. [file 41598_2022_15924_MOESM2_ESM.docx]

Table S1A: Study groups based on reported lifestyle habits in the survey (n=274).

|  |  |  | **Female** | **Male** | **Total** |
| --- | --- | --- | --- | --- | --- |
|  |  |  | (n=188) | (n=81) | (n=269) |
| **Lifestyle habits** | **Question** | **Answer** |  |  | (n/a=5) |
|  |  |  |  |  |  |
| Vitamin supplements | All kinds of vitamins | *Intake* | 91 | 48 | 139 |
|  |  | *Not intake* | 94 | 33 | 127 |
|  |  |  |  |  |  |
|  | Specifically vitamin D | *Intake* | 34(*MC, n=4) | 22(*MC, n=4) | 82(*MC, n=8) |
|  |  | *Not intake* | 156 | 75 | 236 |
|  |  |  |  |  |  |
| Smoking habits | Smoking | *High* | 2 | 2 | 4 |
|  |  | *Intermediate* | 4 | 3 | 7 |
|  |  | *Low* | 68 | 38 | 106 |
|  |  | *None* | 110 | 39 | 149 |
|  |  |  |  |  |  |
|  |  |  |  |  |  |
| Alcohol habits | Alcohol | *High* | 4 | 5 | 9 |
|  | (SD/week) | *Intermediate* | 84 | 45 | 129 |
|  |  | *Low* | 98 | 32 | 130 |
|  |  |  |  |  |  |
|  | Alcohol | *Low* | 31 | 13 | 44 |
|  | (frequency/week) | *High* | 156 | 68 | 224 |
|  |  |  |  |  |  |
|  |  |  |  |  |  |
| Physical activity | Physical activity | *High* | 12 | 7 | 19 |
|  | during summer | *Intermediate* | 95 | 28 | 123 |
|  |  | *Low* | 83 | 47 | 130 |
|  |  |  |  |  |  |
|  | Physical activity | *High* | 20 | 8 | 28 |
|  | during winter | *Intermediate* | 44 | 20 | 64 |
|  |  | *Low* | 126 | 53 | 179 |
|  |  |  |  |  |  |
|  | Physical activity | *High* | 22 | 9 | 31 |
|  | during summer | *Intermediate* | 80 | 25 | 105 |
|  | and winter | *Low* | 88 | 47 | 135 |
|  |  |  |  |  |  |

* n: number of samples, n/a: no answer, MC: Multivitamin complex containing vitamin D

Table S1B: Study groups based on reported lifestyle habits in the survey (n=274).

|  |  |  | **Female** | **Male** | **Total** |
| --- | --- | --- | --- | --- | --- |
|  |  |  | (n=188) | (n=81) | (n=269) |
| **Lifestyle habits** | **Question** | **Answer** |  |  | (n/a=5) |
|  |  |  |  |  |  |
| Sunbathing habits | Sun exposure | *High* | 95 | 32 | 127 |
|  |  | *Intermediate* | 60 | 32 | 92 |
|  |  | *Low* | 33 | 17 | 50 |
|  |  |  |  |  |  |
|  | Use of sunscreen | *Always* | 26 | 30 | 56 |
|  |  | *Sometimes* | 109 | 41 | 150 |
|  |  | *Never* | 52 | 11 | 63 |
|  |  |  |  |  |  |
|  |  |  |  |  |  |
| Eating habits | Fish and | *High* | 21 | 4 | 25 |
|  | seafood (frequency) | *Intermediate* | 118 | 58 | 176 |
|  |  | *Low* | 46 | 20 | 66 |
|  |  |  |  |  |  |

* n: number of samples, n/a: no answer

Table S2: Vitamin D related genes selected for further analyses of association between methylation levels and the study groups

| **Description** | **Source** | **Symbol** | **Nº of probes** |
| --- | --- | --- | --- |
| Vitamin D receptors, metabolic enzymes and transporters | Saponaro et al., 2020  Jang et al., 2019  Bikle, 2014 |  |  |
|  | | *VDR* | 37 |
|  |  | *PDIA3* | 10 |
|  |  | *PDIA3P* | 5 |
|  |  | *PDIA3P1* | 2 |
|  |  | *CYP2R1* | 7 |
|  |  | *CYP27A1* | 20 |
|  |  | *CYP27B1* | 12 |
|  |  | *RXRA* | 80 |
|  |  | *DBP* | 12 |
|  |  | *TRPV6* | 24 |
|  |  | *CYP24A1* | 26 |
| Control of gene expression by vitamin D receptor pathway | Biocarta |  |  |
|  | | *ACTL6A* | 14 |
|  |  | *ARID1A* | 39 |
|  |  | *BAZ1B* | 23 |
|  |  | *CARM1* | 27 |
|  |  | *CHAF1A* | 13 |
|  |  | *COPS2* | 13 |
|  |  | *CREBBP* | 81 |
|  |  | *EP300* | 14 |
|  |  | *HDAC1* | 23 |
|  |  | *KAT2B* | 24 |
|  |  | *MED1* | 7 |
|  |  | *NCOA1* | 27 |
|  |  | *NCOA2* | 76 |
|  |  | *NCOA3* | 46 |
|  |  | *NCOR1* | 37 |
|  |  | *PRMT1* | 22 |
|  |  | *RXRA* | 80 |
|  |  | *SMARCA4* | 56 |
|  |  | *SMARCC1* | 56 |
|  |  | *SMARCC2* | 10 |
|  |  | *SMARCD1* | 14 |
|  |  | *SMARCE1* | 17 |
|  |  | *SNW1* | 14 |
|  |  | *SUPT16H* | 13 |
|  |  | *TOP2B* | 20 |
|  |  | *TSC2* | 80 |

| Primary vitamin D target genes of human monocytes | Nurminen et al.,2019 |  |  |
| --- | --- | --- | --- |
|  | | *SLC25A24* | 24 |
|  |  | *CAMP* | 7 |
|  |  | *CYP24A1* | 26 |
|  |  | *CD14* | 9 |
|  |  | *ITGAM* | 23 |
|  |  | *ALOX5* | 49 |
|  |  | *FANCE* | 8 |
|  |  | *HBEGF* | 10 |
|  |  | *NFKBIA* | 15 |
|  |  | *PDCD1LG2* | 18 |
|  |  | *TMEM37* | 16 |
|  |  | *BHLHE40* | 10 |
|  |  | *CD93* | 15 |
|  |  | *DUSP10* | 21 |
|  |  | *FHL1* | 48 |
|  |  | *MYO7B* | 56 |
|  |  | *PCTP* | 10 |
|  |  | *PPARGC1B* | 41 |
|  |  | *RTCB* | 4 |
|  |  | *SLC37A2* | 29 |
|  |  | *SPI1* | 24 |
|  |  | *AGPAT1* | 72 |
|  |  | *CLMN* | 77 |
|  |  | *COQ3* | 13 |
|  |  | *DND1* | 10 |
|  |  | *NDUFA2* | 14 |
|  |  | *PSMB1* | 16 |
|  |  | *SLC25A15* | 10 |
|  |  | *SLC52A2* | 7 |
|  |  | *TBP* | 8 |
|  |  | *TMCO6* | 15 |
|  |  | *WDR55* | 14 |
|  |  | *ZNF44* | 16 |
|  |  | *ACSL1* | 64 |
|  |  | *CDA* | 18 |
|  |  | *FBP1* | 16 |
|  |  | *G0S2* | 12 |
|  |  | *GLIPR1* | 15 |
|  |  | *INSR* | 57 |
|  |  | *ITNS1* | 58 |
|  |  | *KLHDC8B* | 11 |
|  |  | *PNPLA1* | 30 |
|  |  | *SERINC2* | 27 |
|  |  | *SOAT1* | 15 |
|  |  | *SSH1* | 77 |
