# Supplementary material for: Characterization of methylation patterns associated with lifestyle factors and vitamin D supplementation in a healthy elderly cohort from Southwest Sweden
Source: Sci Rep. 2022 Jul 25;12:12670. doi: 10.1038/s41598-022-15924-x (PMC9310683; doi:10.1038/s41598-022-15924-x)
Supplement: Supplementary file 3 — Supplementary Information 3. [file 41598_2022_15924_MOESM3_ESM.docx]

Table S3: DMPs found for the comparison between high smoking (n=4) and not smoking (n=149).

| Gene | Probe | chr | fdr | β |
| --- | --- | --- | --- | --- |
| PRKCSH, CCDC151 | cg17013823 | 19 | 6.3E-11 | 0.24 |
| TMEM161B | cg24720694 | 5 | 1.7E-07 | -0.25 |
| NSMCE2 | cg10741696 | 8 | 5.8E-07 | -0.27 |
| Intergenic region | cg01481906 | 9 | 4.4E-06 | -0.23 |
| USP10 | cg11193354 | 16 | 5.3E-06 | 0.30 |
| SEC24A | cg20879468 | 5 | 7.6E-05 | 0.21 |
| Intergenic region | cg16395282 | 1 | 0.0002 | -0.20 |
| G6PC | cg10475574 | 17 | 0.0004 | -0.20 |
| ACSS1 | cg03519012 | 20 | 0.006 | 0.21 |
| Intergenic region | cg06624310 | 2 | 0.03 | 0.20 |
| CD81 | cg03848831 | 11 | 0.04 | -0.21 |

chr: Chromosome; fdr: False discovery rate and β: beta value difference.

Table S4: DMPs found for both the comparison between the group taking vitamin D supplementation in a MC (n=8) with the group not taking vitamin D (n=229); and with the group taking vitamin D not in a MC (n=33).

| Gene | Probe | chr | Fdr | β |
| --- | --- | --- | --- | --- |
| *SLC25A24* | cg05918124 | 1 | 0.03 | 0.20 |

chr: Chromosome; fdr: False discovery rate and β: beta value difference.

Table S5: DMPs for the comparison of reported moderate-high physical activity (n= 262) with very low or almost no physical activity (n=7).

| Gene | Probe | chr | Fdr | β |
| --- | --- | --- | --- | --- |
| Intergenic region | cg11671940 | 10 | 0,004 | -0,29 |
| Intergenic region | cg05141217 | 8 | 0,027 | -0,23 |

chr: Chromosome; fdr: False discovery rate and diff: β value difference.

Table S6: DMPs found for the comparison of the vitamin D intake and High exercise group (n=7) with the group with low exercise and not vitamin D intake (n=118).

| Gene | Probe | chr | fdr | β |
| --- | --- | --- | --- | --- |
| DAB2 | cg21886364 | 5 | 6.5E-07 | 0.28 |
| COL23A1 | cg07145979 | 5 | 7E-07 | 0.28 |
| SOD3 | cg17573292 | 4 | 2.7E-06 | 0.34 |
| SKI | cg13488570 | 1 | 4.5E-06 | 0.23 |
| LPIN1 | cg00523161 | 2 | 4.5E-06 | 0.34 |
| Intergenic region | cg24792289 | 5 | 4.5E-06 | 0.27 |
| Intergenic region | cg23244910 | 6 | 6.9E-06 | 0.28 |
| GLI3 | cg06310816 | 7 | 2.1E-05 | 0.20 |
| LOC100132111, C2CD4D | cg26426745 | 1 | 2.4E-05 | 0.35 |
| RPH3AL | cg15295273 | 17 | 2.4E-05 | 0.31 |
| RTN4RL1 | cg08454053 | 17 | 3.2E-05 | 0.24 |
| KIAA0319L | cg22698544 | 1 | 7.1E-05 | 0.26 |
| Intergenic region | cg05964935 | Y | 7.1E-05 | -0.29 |
| NKX2-8 | cg20008148 | 14 | 8.8E-05 | 0.36 |
| NTM | cg04934246 | 11 | 2.3E-04 | 0.21 |
| Intergenic region | cg16668359 | 2 | 2.3E-04 | 0.31 |
| NFIX | cg20116828 | 19 | 3E-04 | 0.38 |
| Intergenic region | cg20586840 | 4 | 3.3E-04 | -0.21 |
| IQSEC1 | cg16562217 | 3 | 3.7E-04 | 0.56 |
| Intergenic region | cg13100965 | 7 | 3.8E-04 | 0.31 |
| ITGB4 | cg16916914 | 17 | 4.2E-04 | 0.38 |
| PKN1 | cg00880290 | 19 | 4.4E-04 | 0.21 |
| SGK1 | cg08550353 | 6 | 4.7E-04 | 0.25 |
| DPH3B, TCFL5 | cg17371014 | 20 | 4.8E-04 | 0.32 |
| FAM43A | cg09652652 | 3 | 5.1E-04 | 0.21 |
| Intergenic region | cg19623418 | 20 | 5.2E-04 | 0.26 |
| WDR66 | cg21171335 | 12 | 5.2E-04 | 0.23 |
| FNDC1 | cg09107912 | 6 | 5.6E-04 | 0.24 |
| MPRIP | cg19342711 | 17 | 6.1E-04 | -0.41 |
| Intergenic region | cg01064683 | 2 | 6.35E-04 | 0.20 |
| MAP3K6 | cg20996351 | 1 | 7.4E-04 | 0.26 |
| SEMA6B | cg25806655 | 19 | 7.4E-04 | 0.23 |
| WNT7B | cg22413388 | 22 | 7.4E-04 | 0.24 |
| KLHDC7B | cg03957481 | 22 | 8.4E-04 | 0.25 |
| AARD | cg18274779 | 8 | 1E-03 | -0.41 |
| HHEX | cg00487187 | 10 | 1E-03 | 0.29 |
| SLC34A2 | cg09157302 | 4 | 1.2E-03 | 0.24 |
| DLX1 | cg17737681 | 2 | 1.2E-03 | 0.22 |
| ARPIN, C15orf38-AP3S2 | cg25755215 | 15 | 1.2E-03 | -0.22 |
| OTX1 | cg21472506 | 2 | 1.3E-03 | 0.38 |
| PARP15 | cg23442853 | 3 | 1.3E-03 | 0.23 |
| Intergenic region | cg03570708 | 5 | 1.3E-03 | 0.29 |
| STAP2 | cg14263858 | 19 | 1.3E-03 | 0.28 |
| Intergenic region | cg05262224 | 16 | 1.4E-03 | 0.22 |
| WDR66 | cg11867697 | 12 | 1.4E-03 | 0.24 |
| ZDHHC1, TPPP3 | cg13973436 | 16 | 1.5E-03 | 0.22 |
| ZNF639 | cg24102349 | 3 | 1.5E-03 | -0.20 |
| Intergenic region | cg21082028 | 17 | 1.6E-03 | 0.21 |
| BLCAP | cg01402569 | 20 | 1.6E-03 | -0.36 |
| Intergenic region | cg11478200 | 6 | 1.6E-03 | 0.31 |
| Intergenic region | cg19746541 | 3 | 1.6E-03 | 0.22 |
| SGIP1 | cg02641990 | 1 | 1.8E-03 | 0.24 |
| Intergenic region | cg00604454 | 16 | 1.8E-03 | 0.24 |
| CDK2AP1 | cg20181887 | 12 | 1.9E-03 | 0.21 |
| UROS | cg09038666 | 10 | 1.9E-03 | 0.22 |
| Intergenic region | cg27122441 | 22 | 1.9E-03 | 0.20 |
| CDH3 | cg06575065 | 16 | 1.9E-03 | 0.22 |
| Intergenic region | cg10511890 | 11 | 2E-03 | 0.35 |
| FAM110B | cg23881926 | 8 | 2E-03 | 0.29 |
| SFXN5 | cg10051217 | 2 | 2.1E-03 | 0.35 |
| PIK3AP1 | cg27190138 | 10 | 2.1E-03 | 0.28 |
| GADL1 | cg18005693 | 3 | 2.1E-03 | -0.23 |
| RFX5 | cg26796396 | 1 | 2.2E-03 | -0.34 |
| Intergenic region | cg21590108 | 8 | 2.3E-03 | -0.20 |
| C21orf70 | cg11327657 | 21 | 2.3E-03 | 0.25 |
| ODZ4 | cg17542408 | 11 | 2.3E-03 | 0.23 |
| EGFL7 | cg03023837 | 9 | 2.4E-03 | 0.26 |
| ODZ4 | cg12162138 | 11 | 2.5E-03 | 0.25 |
| PRKCH | cg09945745 | 14 | 2.5E-03 | 0.20 |
| SLC43A2 | cg09329516 | 17 | 2.6E-03 | -0.23 |
| NEURL4 | cg26889226 | 17 | 2.6E-03 | 0.20 |
| TICAM2, LOC101927100 | cg10472270 | 5 | 2.7E-03 | -0.20 |
| DYSF | cg21165262 | 2 | 2.7E-03 | 0.25 |
| PRKAR1B | cg25495650 | 7 | 2.9E-03 | 0.27 |
| PMEPA1 | cg26681770 | 20 | 2.9E-03 | 0.40 |
| Intergenic region | cg21729992 | 20 | 3E-03 | 0.25 |
| DNMT1 | cg09445675 | 19 | 3.1E-03 | 0.21 |
| DLX6AS | cg03774463 | 7 | 3.2E-03 | 0.20 |
| CRACR2A | cg04614981 | 12 | 3.2E-03 | 0.22 |
| C17orf56 | cg24433586 | 17 | 3.3E-03 | 0.24 |
| ARRDC2 | cg23482747 | 19 | 3.4E-03 | 0.23 |
| PFDN1 | cg25409734 | 5 | 3.4E-03 | -0.40 |
| MARC1 | cg01219672 | 1 | 3.5E-03 | 0.22 |
| TIMELESS | cg14718370 | 12 | 3.5E-03 | 0.20 |
| ZNF782 | cg14176626 | 9 | 3.6E-03 | 0.22 |
| SDSL | cg08314252 | 12 | 3.6E-03 | 0.22 |
| TMED7-TICAM2 | cg22173752 | 5 | 3.7E-03 | -0.21 |
| CDH3 | cg00748373 | 16 | 3.8E-03 | 0.26 |
| HPS4 | cg16170930 | 22 | 3.8E-03 | -0.20 |
| BEND4 | cg23951961 | 4 | 4.1E-03 | 0.21 |
| NUAK1 | cg20060685 | 12 | 4.2E-03 | 0.21 |
| S1PR4 | cg20695297 | 19 | 4.4E-03 | 0.23 |
| TMEM163 | cg18712599 | 2 | 4.4E-03 | 0.32 |
| Intergenic region | cg08838158 | 2 | 4.5E-03 | 0.27 |
| Intergenic region | cg00210271 | 12 | 4.6E-03 | 0.21 |
| ARID3A | cg08553572 | 19 | 4.6E-03 | 0.21 |
| NKX1-2 | cg08932440 | 10 | 4.7E-03 | 0.20 |
| NR2F1 | cg17774851 | 5 | 5E-03 | 0.23 |
| NUAK1 | cg18525352 | 12 | 5.2E-03 | 0.22 |
| ARID5A | cg19954537 | 2 | 5.2E-03 | 0.20 |
| TFCP2 | cg20392585 | 12 | 5.2E-03 | -0.20 |
| Intergenic region | cg10293403 | 6 | 5.2E-03 | 0.28 |
| SLC38A10 | cg16863795 | 17 | 5.2E-03 | 0.28 |
| ACCN4 | cg25350252 | 2 | 5.3E-03 | -0.22 |
| WNT6 | cg00011225 | 2 | 5.3E-03 | 0.28 |
| SPI1 | cg15982099 | 11 | 5.5E-03 | 0.31 |
| Intergenic region | cg08144675 | 16 | 5.5E-03 | 0.29 |
| Intergenic region | cg00833524 | 6 | 5.7E-03 | -0.21 |
| CSRNP3 | cg22674796 | 2 | 5.8E-03 | 0.29 |
| Intergenic region | cg06797478 | 13 | 6E-03 | 0.20 |
| COL25A1 | cg22548304 | 4 | 6.4E-03 | -0.23 |
| TCERG1L | cg11844537 | 10 | 6.4E-03 | 0.25 |
| LRP5 | cg12895546 | 11 | 6.4E-03 | 0.24 |
| PLAGL1 | cg02279224 | 6 | 6.4E-03 | -0.33 |
| PEAR1 | cg19260376 | 1 | 6.5E-03 | 0.28 |
| Intergenic region | cg24305906 | 2 | 6.5E-03 | 0.34 |
| ZDHHC1 | cg09451235 | 16 | 6.5E-03 | 0.21 |
| Intergenic region | cg16820798 | 11 | 6.7E-03 | 0.24 |
| TBX1 | cg04999026 | chr22 | 6.8E-03 | 0.24 |
| Intergenic region | cg06207801 | chr7 | 6.8E-03 | 0.21 |
| Intergenic region | cg00851389 | chr16 | 6.8E-03 | 0.20 |
| Intergenic region | cg10932018 | chr5 | 6.9E-03 | 0.21 |
| PROCA1 | cg18448746 | chr17 | 6.9E-03 | 0.26 |
| DAZL | cg06648556 | chr3 | 6.9E-03 | 0.39 |
| NFATC4 | cg24785555 | chr14 | 7E-03 | 0.23 |
| TBX1 | cg01697719 | chr22 | 7E-03 | 0.20 |
| GPC6 | cg00191477 | chr13 | 7.1E-03 | -0.39 |
| AADACL4 | cg01040782 | 1 | 7.3E-03 | 0.31 |
| ATXN2L | cg25935597 | 16 | 7.4E-03 | 0.22 |
| Intergenic region | cg05793579 | 16 | 7.4E-03 | 0.23 |
| Intergenic region | cg24105287 | 2 | 7.4E-03 | 0.29 |
| Intergenic region | cg01889143 | 5 | 7.4E-03 | 0.27 |
| STAP2 | cg19058865 | 19 | 7.5E-03 | 0.32 |
| CADM1 | cg13790125 | 11 | 7.5E-03 | 0.21 |
| MAML3 | cg00875805 | 4 | 7.6E-03 | 0.22 |
| BTBD12 | cg02950621 | 16 | 8E-03 | 0.20 |
| RASL10A | cg12552771 | 22 | 8E-03 | 0.23 |
| HPS3 | cg12108375 | 3 | 8.1E-03 | -0.21 |
| Intergenic region | cg16917697 | 6 | 8.2E-03 | -0.26 |
| LGALS8 | cg19176897 | 1 | 8.3E-03 | -0.22 |
| Intergenic region | cg15334372 | 16 | 8.3E-03 | 0.21 |
| WNT6 | cg22587479 | 2 | 8.4E-03 | 0.31 |
| TTTY14 | cg17570153 | Y | 8.5E-03 | -0.31 |
| FOXI1 | cg16087093 | 5 | 8.6E-03 | 0.23 |
| FLJ37453 | cg01429859 | 1 | 8.8E-03 | 0.30 |
| HHEX | cg26979504 | 10 | 8.8E-03 | 0.21 |
| METTL20 | cg10415216 | 12 | 8.9E-03 | -0.41 |
| Intergenic region | cg24788157 | 10 | 9.1E-03 | 0.21 |
| SCARA5 | cg12116192 | 8 | 9.2E-03 | -0.21 |
| ATP4A | cg19903766 | 19 | 9.5E-03 | -0.20 |
| JAK3 | cg05635754 | 19 | 9.6E-03 | 0.21 |
| FAM110B | cg01602153 | 8 | 9.7E-03 | 0.28 |
| Intergenic region | cg09947501 | 6 | 9.9E-03 | -0.22 |
| SARM1 | cg25356504 | 17 | 0.01 | 0.22 |
| Intergenic region | cg19594252 | Y | 0.01 | -0.24 |
| TFCP2 | cg11297723 | 12 | 0.01 | -0.21 |
| Intergenic region | cg15815333 | 10 | 0.01 | 0.21 |
| MFSD10 | cg17653203 | 4 | 0.01 | 0.23 |
| Intergenic region | cg18054755 | 12 | 0.01 | -0.28 |
| EMILIN1 | cg02804722 | 2 | 0.01 | 0.27 |
| TFAP2E | cg07167423 | 1 | 0.01 | 0.25 |
| STAP2 | cg25528916 | 19 | 0.01 | 0.32 |
| TTTY5 | cg06865724 | Y | 0.01 | -0.26 |
| AGRN | cg27541454 | 1 | 0.01 | 0.29 |
| NFIX | cg06623219 | 19 | 0.01 | 0.36 |
| HRH1 | cg12246177 | 3 | 0.01 | -0.21 |
| Intergenic region | cg19529472 | 8 | 0.01 | -0.33 |
| SLC17A9 | cg19142181 | 20 | 0.01 | 0.23 |
| TKT | cg07621169 | 3 | 0.01 | -0.21 |
| Intergenic region | cg08463633 | 8 | 0.01 | -0.30 |
| TBX1 | cg08382235 | 22 | 0.01 | 0.25 |
| LOC339524 | cg02083836 | 1 | 0.01 | 0.31 |
| EPB49 | cg02046552 | 8 | 0.01 | 0.29 |
| Intergenic region | cg15016740 | 5 | 0.01 | 0.21 |
| Intergenic region | cg05524765 | X | 0.01 | -0.23 |
| ITGAE | cg13984928 | 17 | 0.01 | 0.22 |
| Intergenic region | cg01093369 | 5 | 0.01 | 0.30 |
| TMEM30B | cg01835384 | 14 | 0.01 | -0.20 |
| TOX3 | cg06758255 | 16 | 0.01 | 0.21 |
| CTBP2 | cg23000734 | 10 | 0.01 | 0.26 |
| Intergenic region | cg03264133 | 6 | 0.01 | -0.25 |
| Intergenic region | cg01301803 | 10 | 0.01 | 0.22 |
| Intergenic region | cg13200556 | 8 | 0.01 | -0.21 |
| DRC1 | cg20327067 | 2 | 0.01 | -0.21 |
| Intergenic region | cg06919231 | 6 | 0.01 | 0.27 |
| Intergenic region | cg01615050 | 6 | 0.01 | -0.47 |
| Intergenic region | cg21215550 | 9 | 0.01 | 0.25 |
| CLYBL | cg12790874 | 13 | 0.01 | 0.21 |
| Intergenic region | cg13261971 | 2 | 0.02 | 0.23 |
| SCARF1 | cg00869668 | 17 | 0.02 | 0.21 |
| Intergenic region | cg24808105 | 5 | 0.02 | 0.22 |
| Intergenic region | cg08217163 | 2 | 0.02 | 0.26 |
| NXPH4 | cg22215815 | 12 | 0.02 | 0.26 |
| GSTA4 | cg17046180 | 6 | 0.02 | 0.23 |
| CLYBL | cg03041920 | 13 | 0.02 | 0.22 |
| Intergenic region | cg19107647 | 18 | 0.02 | -0.20 |
| Intergenic region | cg08985767 | 9 | 0.02 | -0.21 |
| C9orf122 | cg21189438 | 9 | 0.02 | 0.25 |
| SERPINB13 | cg14260304 | 18 | 0.02 | -0.23 |
| MMD2 | cg13009011 | 7 | 0.02 | 0.25 |
| ANO1 | cg11157765 | 11 | 0.02 | 0.23 |
| KLHL6 | cg16819999 | 3 | 0.02 | 0.21 |
| Intergenic region | cg04554929 | 8 | 0.02 | 0.33 |
| EPS8L2 | cg08327690 | 11 | 0.02 | 0.22 |
| SLC1A2 | cg10730349 | 11 | 0.02 | 0.28 |
| SPTLC2 | cg18872420 | 14 | 0.02 | 0.49 |
| Intergenic region | cg00367632 | 11 | 0.02 | -0.38 |
| GPR85 | cg06369352 | 7 | 0.02 | -0.23 |
| Intergenic region | cg08149869 | 21 | 0.02 | -0.20 |
| Intergenic region | cg08149869 | 21 | 0.02 | -0.20 |
| CACNB2 | cg12657758 | 10 | 0.02 | -0.23 |
| RERE | cg23963229 | 1 | 0.02 | 0.25 |
| THSD7B | cg16561226 | 2 | 0.02 | -0.23 |
| PHLDB2 | cg25711003 | 3 | 0.02 | -0.20 |
| NOX4 | cg03793270 | 11 | 0.02 | 0.25 |
| ZNF276, VPS9D1 | cg23973564 | 16 | 0.02 | 0.27 |
| TBC1D3F | cg24141001 | 17 | 0.02 | 0.31 |
| Intergenic region | cg24751773 | 2 | 0.02 | -0.20 |
| Intergenic region | cg02034689 | 5 | 0.02 | 0.20 |
| WIPI2 | cg17652756 | 7 | 0.02 | 0.21 |
| Intergenic region | cg13519035 | 12 | 0.02 | 0.22 |
| SLC12A8 | cg26125625 | 3 | 0.02 | 0.24 |
| CCL20 | cg08575688 | 2 | 0.02 | -0.20 |
| Intergenic region | cg00658440 | 12 | 0.02 | -0.20 |
| PARP15 | cg21974923 | 3 | 0.02 | 0.22 |
| GNA12 | cg26247093 | 7 | 0.02 | 0.20 |
| Intergenic region | cg00790756 | 20 | 0.02 | 0.24 |
| TRH | cg27444828 | 3 | 0.02 | 0.32 |
| TRAP1 | cg12359832 | 16 | 0.02 | 0.53 |
| LYPD1 | cg08613144 | 2 | 0.02 | 0.25 |
| RUNX1 | cg11498607 | 21 | 0.02 | 0.20 |
| Intergenic region | cg09532371 | 1 | 0.02 | 0.33 |
| Intergenic region | cg26402801 | 4 | 0.02 | 0.21 |
| Intergenic region | cg06839896 | 12 | 0.02 | 0.23 |
| XRCC5 | cg05264639 | 2 | 0.02 | -0.20 |
| SLC1A2 | cg16029801 | 11 | 0.02 | 0.32 |
| SOD3 | cg13096007 | 4 | 0.02 | 0.21 |
| PIGG, ZNF721 | cg09271316 | 4 | 0.02 | -0.21 |
| TFCP2 | cg22790973 | 12 | 0.02 | -0.21 |
| Intergenic region | cg18096962 | 5 | 0.02 | 0.24 |
| Intergenic region | cg01020413 | 2 | 0.02 | 0.24 |
| PTPRU | cg00914222 | 1 | 0.02 | 0.25 |
| Intergenic region | cg00349404 | 10 | 0.02 | -0.23 |
| Intergenic region | cg18085787 | Y | 0.02 | 0.31 |
| SLC41A1 | cg09064754 | 1 | 0.02 | -0.28 |
| MTA1 | cg16015362 | 14 | 0.02 | -0.28 |
| LOC100132111, C2CD4D | cg24051554 | 1 | 0.02 | 0.29 |
| Intergenic region | cg18635432 | 14 | 0.02 | -0.24 |
| TRIM58 | cg26157385 | 1 | 0.02 | 0.20 |
| DNAJC24 | cg19738233 | 11 | 0.03 | 0.38 |
| CSMD3 | cg14649914 | 8 | 0.03 | -0.41 |
| TSNAX, TSNAX-DISC1. | cg08020573 | 1 | 0.03 | 0.21 |
| Intergenic region | cg26510033 | 10 | 0.03 | 0.21 |
| Intergenic region | cg19801262 | 1 | 0.03 | 0.25 |
| LHX5 | cg26843324 | 12 | 0.03 | 0.21 |
| Intergenic region | cg04880105 | 4 | 0.03 | -0.20 |
| Intergenic region | cg03517284 | 6 | 0.03 | -0.29 |
| FOSB | cg12542255 | 19 | 0.03 | 0.22 |
| ARHGEF10 | cg24138325 | 8 | 0.03 | 0.21 |
| SHPRH | cg23082221 | 6 | 0.03 | 0.22 |
| TCHH | cg05523911 | 1 | 0.03 | 0.21 |
| ANKLE2 | cg23075597 | 12 | 0.03 | 0.40 |
| Intergenic region | cg08311646 | 7 | 0.03 | -0.21 |
| Intergenic region | cg25215230 | 4 | 0.03 | -0.22 |
| MC3R | cg20553766 | 20 | 0.03 | -0.29 |
| GADL1 | cg03629794 | 3 | 0.03 | -0.24 |
| PALLD | cg22587857 | 4 | 0.03 | -0.23 |
| NXNL2 | cg24917037 | 9 | 0.03 | -0.26 |
| Intergenic region | cg20463151 | 8 | 0.03 | -0.26 |
| STARD13 | cg07499182 | 13 | 0.03 | 0.29 |
| CDH22 | cg17771031 | 20 | 0.03 | 0.26 |
| ICAM4 | cg00389463 | 19 | 0.03 | 0.24 |
| Intergenic region | cg02326806 | 15 | 0.03 | 0.26 |
| Intergenic region | cg16481612 | 12 | 0.03 | -0.21 |
| Intergenic region | cg25371919 | 2 | 0.03 | 0.20 |
| Intergenic region | cg19285800 | 13 | 0.03 | 0.21 |
| Intergenic region | cg23399379 | 4 | 0.03 | -0.21 |
| Intergenic region | cg14883682 | 5 | 0.03 | -0.22 |
| INPP5D | cg00438740 | 2 | 0.03 | 0.23 |
| CRISPLD2 | cg26709988 | 16 | 0.03 | 0.20 |
| OTX1 | cg23229261 | 2 | 0.03 | 0.31 |
| PARP1 | cg24937136 | 1 | 0.03 | 0.21 |
| GSDMA | cg21230266 | 17 | 0.03 | -0.24 |
| TRAP1 | cg04681450 | 16 | 0.03 | 0.43 |
| LCA5 | cg02841199 | 6 | 0.03 | -0.25 |
| Intergenic region | cg05224741 | 6 | 0.03 | 0.24 |
| MBP | cg25389087 | 18 | 0.03 | 0.22 |
| C11orf20 | cg18383813 | 11 | 0.03 | -0.28 |
| TCERG1L | cg11872966 | 10 | 0.03 | 0.21 |
| TARS | cg01708290 | 5 | 0.04 | 0.35 |
| SNX19 | cg02624129 | 11 | 0.04 | 0.35 |
| Intergenic region | cg03522146 | 5 | 0.04 | -0.21 |
| Intergenic region | cg11442918 | 12 | 0.04 | -0.20 |
| TMEM30B | cg24785368 | 14 | 0.04 | -0.21 |
| STAT5A | cg14042635 | 17 | 0.04 | 0.27 |
| TCERG1L | cg08617581 | 10 | 0.04 | 0.24 |
| C2CD4D, LOC100132111 | cg10781408 | 1 | 0.04 | 0.21 |
| Intergenic region | cg06973615 | 2 | 0.04 | 0.21 |
| DLX5 | cg20377305 | 7 | 0.04 | 0.21 |
| Intergenic region | cg04031201 | 4 | 0.04 | -0.26 |
| RSPH6A, SYMPK | cg02505812 | 19 | 0.04 | 0.24 |
| CACNA1A | cg22304399 | 19 | 0.04 | 0.24 |
| SORBS2 | cg14516100 | 4 | 0.04 | 0.32 |
| FAM118B | cg06675602 | 11 | 0.04 | -0.22 |
| COLEC11 | cg10403091 | 2 | 0.04 | 0.20 |
| OR8J3 | cg05168668 | 11 | 0.04 | -0.20 |
| MTMR3 | cg04740981 | 22 | 0.04 | -0.21 |
| Intergenic region | cg25923609 | 5 | 0.04 | 0.26 |
| ANO1-AS2 | cg13449673 | 11 | 0.04 | 0.22 |
| ZNF257 | cg17555429 | 19 | 0.04 | -0.21 |
| C14orf4 | cg13493526 | 14 | 0.04 | -0.20 |
| RADIL | cg04314711 | 7 | 0.04 | 0.23 |
| TIMP2 | cg05306745 | 17 | 0.04 | 0.23 |
| Intergenic region | cg18261205 | 15 | 0.04 | 0.21 |
| HLA-DRB5 | cg26981746 | 6 | 0.04 | -0.30 |
| LYPD5 | cg12768605 | 19 | 0.04 | -0.23 |
| RNF39 | cg13918754 | 6 | 0.04 | 0.29 |
| OPLAH | cg17301223 | 8 | 0.04 | 0.27 |
| Intergenic region | cg15084585 | 8 | 0.04 | 0.36 |
| COL25A1 | cg09902898 | 4 | 0.04 | -0.22 |
| ENOSF1 | cg07100532 | 18 | 0.04 | -0.22 |
| SPEG | cg25925023 | 2 | 0.04 | 0.23 |
| SEC16B | cg00647232 | 1 | 0.04 | 0.21 |
| Intergenic region | cg13208088 | 6 | 0.04 | 0.23 |
| Intergenic region | cg20168806 | 6 | 0.04 | 0.21 |
| HOXD13 | cg04415176 | 2 | 0.04 | 0.21 |
| WDR27 | cg14691985 | 6 | 0.04 | 0.34 |
| Intergenic region | cg07474083 | 16 | 0.04 | -0.22 |
| Intergenic region | cg22973468 | 8 | 0.04 | 0.21 |
| Intergenic region | cg24452128 | 6 | 0.04 | 0.32 |
| Intergenic region | cg23681866 | 6 | 0.04 | -0.36 |
| COL18A1 | cg20383948 | 21 | 0.04 | 0.28 |
| PPP1R13L | cg27152890 | 19 | 0.04 | 0.26 |
| OR6P1 | cg25280720 | 1 | 0.04 | 0.37 |
| ZIC1 | cg02519751 | 3 | 0.04 | 0.21 |
| PDE11A | cg24252907 | 2 | 0.04 | -0.32 |
| Intergenic region | cg02513556 | 12 | 0.04 | 0.23 |
| ENOSF1 | cg10004653 | 18 | 0.04 | -0.25 |
| CLIC3 | cg26815843 | 9 | 0.04 | -0.21 |
| Intergenic region | cg11089122 | 1 | 0.04 | 0.23 |
| DOCK6 | cg12019614 | 19 | 0.04 | 0.22 |
| Intergenic region | cg08214455 | 16 | 0.04 | 0.21 |
| TAPT1 | cg00555420 | 4 | 0.04 | 0.26 |
| PCDHGA, PCDHGB | cg09639151 | 5 | <0.05 | 0.25 |
| Intergenic region | cg24139837 | X | <0.05 | -0.34 |
| IGSF9 | cg11950939 | 1 | <0.05 | -0.20 |
| Intergenic region | cg20093123 | 13 | <0.05 | -0.21 |
| LMO7 | cg10726445 | 13 | <0.05 | -0.22 |
| TRAP1 | cg08842287 | 16 | <0.05 | -0.20 |
| Intergenic region | cg17754510 | 6 | <0.05 | 0.23 |
| OSBP2 | cg00607058 | 22 | <0.05 | 0.21 |
| RBM38 | cg27262236 | 20 | <0.05 | 0.24 |
| Intergenic region | cg12170574 | 7 | <0.05 | 0.22 |
| Intergenic region | cg09761058 | 17 | <0.05 | -0.41 |
| Intergenic region | cg06017490 | 7 | <0.05 | 0.22 |
| Intergenic region | cg01557799 | 11 | <0.05 | 0.20 |
| RUNX3 | cg21406271 | 1 | <0.05 | -0.21 |

chr: Chromosome; fdr: False discovery rate and diff: β value difference.

Table S7: DMRs found for the comparison of vitamin D intake and high exercise group (n=7) with the group with low exercise and not vitamin D intake (n=118).

| Gene | Region | chr | Fisher | diff |
| --- | --- | --- | --- | --- |
| NFIX | DMR1 | chr19 | 5.1E-06 | 0.22 |
| DPYS | DMR2 | chr8 | 3.4E-05 | 0.21 |
| TCERG1L | DMR3 | chr10 | 2.9E-04 | 0.20 |
| SLC17A3, HIST1H2APS2 | DMR4 | chr6 | 3.1E-04 | -0.31 |
| MPRIP | DMR5 | chr17 | 6.7E-04 | -0.26 |
| PMEPA1 | DMR6 | chr20 | 1.7E-03 | 0.20 |
| DNASE1, TRAP1 | DMR7 | chr16 | 5.4E-03 | 0.48 |
| SPTLC2 | DMR8 | chr14 | 9.1E-03 | 0.36 |
| Intergenic region | DMR9 | chr10 | 2.9E-02 | -0.21 |

chr: Chromosome and diff: β value difference.

Table S8: DMPs for the vitamin D intake and high exercise group binding to promoter regions (TSS200 and TSS1500).

| Gene | Probe | Gene region |
| --- | --- | --- |
| AADACL4 | cg01040782 | TSS200 |
| RUNX3 | cg21406271 | TSS1500;Body |
| LOC100132111, C2CD4D | cg26426745 | TSS1500;Body |
| C2CD4D, LOC100132111 | cg10781408 | Body;TSS200 |
| SLC41A1 | cg09064754 | TSS200 |
| LGALS8 | cg19176897 | TSS1500;Body;5'UTR |
| DRC1 | cg20327067 | TSS1500 |
| EMILIN1 | cg02804722 | TSS1500 |
| LYPD1 | cg08613144 | 1stExon;5'UTR;TSS1500 |
| CSRNP3 | cg22674796 | TSS1500 |
| SPEG | cg25925023 | TSS1500 |
| CCL20 | cg08575688 | TSS200;TSS200 |
| INPP5D | cg00438740 | TSS200;TSS200 |
| DAZL | cg06648556 | TSS1500;1stExon;5'UTR |
| GADL1 | cg03629794 | TSS200 |
| PARP15 | cg21974923 | TSS200 |
| TRH | cg27444828 | TSS200 |
| ZIC1 | cg02519751 | TSS1500 |
| HPS3 | cg12108375 | TSS200 |
| ZNF639 | cg24102349 | TSS1500 |
| PIGG, ZNF721 | cg09271316 | 1stExon;5'UTR;TSS1500;Body |
| SLC34A2 | cg09157302 | TSS1500 |
| BEND4 | cg23951961 | TSS200 |
| PFDN1 | cg25409734 | TSS200 |
| LCA5 | cg02841199 | TSS1500 |
| SGK1 | cg08550353 | TSS1500;Body |
| PLAGL1 | cg02279224 | TSS1500 |
| FNDC1 | cg09107912 | TSS1500 |
| WIPI2 | cg17652756 | TSS1500;Body |
| GPR85 | cg06369352 | 5'UTR;TSS1500 |
| EPB49 | cg02046552 | TSS1500;5'UTR |
| CSMD3 | cg14649914 | TSS1500 |
| NXNL2 | cg24917037 | TSS200 |
| ZNF782 | cg14176626 | TSS1500 |
| EGFL7 | cg03023837 | TSS200 |
| CLIC3 | cg26815843 | TSS200 |
| CTBP2 | cg23000734 | TSS1500 |
| SLC1A2 | cg16029801 | TSS1500;TSS200 |
| OR8J3 | cg05168668 | TSS1500 |
| C11orf20 | cg18383813 | TSS200 |
| ANO1-AS2 | cg13449673 | TSS200 |
| NOX4 | cg03793270 | 5'UTR;TSS200 |
| TFCP2 | cg20392585 | TSS200;TSS1500 |
| NUAK1 | cg18525352 | TSS200 |
| WDR66 | cg21171335 | TSS200 |
| GPC6 | cg00191477 | TSS200 |
| NFATC4 | cg24785555 | TSS200;Body |
| NKX2-8 | cg20008148 | TSS1500 |
| TMEM30B | cg24785368 | TSS200 |
| ZDHHC1,TPPP3 | cg13973436 | Body;TSS1500 |
| CDH3 | cg00748373 | TSS1500 |
| ZNF276,VPS9D1 | cg23973564 | TSS1500;Body |
| ITGAE | cg13984928 | TSS200 |
| PROCA1 | cg18448746 | TSS1500 |
| TIMP2 | cg05306745 | TSS1500 |
| ENOSF1 | cg10004653 | TSS1500 |
| ICAM4 | cg00389463 | TSS200 |
| PKN1 | cg00880290 | TSS200;Body |
| ZNF257 | cg17555429 | TSS200 |
| LYPD5 | cg12768605 | TSS200 |
| RSPH6A, SYMPK | cg02505812 | TSS200;3'UTR |
| BLCAP | cg01402569 | TSS200;TSS1500 |
| RBM38 | cg27262236 | TSS1500 |
| DPH3B, TCFL5 | cg17371014 | TSS200;Body |
| HPS4 | cg16170930 | TSS1500; Body |
| OSBP2 | cg00607058 | TSS1500 |
| TTTY5 | cg06865724 | TSS1500 |
