# Supplementary material for: Characterization of methylation patterns associated with lifestyle factors and vitamin D supplementation in a healthy elderly cohort from Southwest Sweden
Source: Sci Rep. 2022 Jul 25;12:12670. doi: 10.1038/s41598-022-15924-x (PMC9310683; doi:10.1038/s41598-022-15924-x)
Supplement: Supplementary file 4 — Supplementary Information 4. [file 41598_2022_15924_MOESM4_ESM.docx]

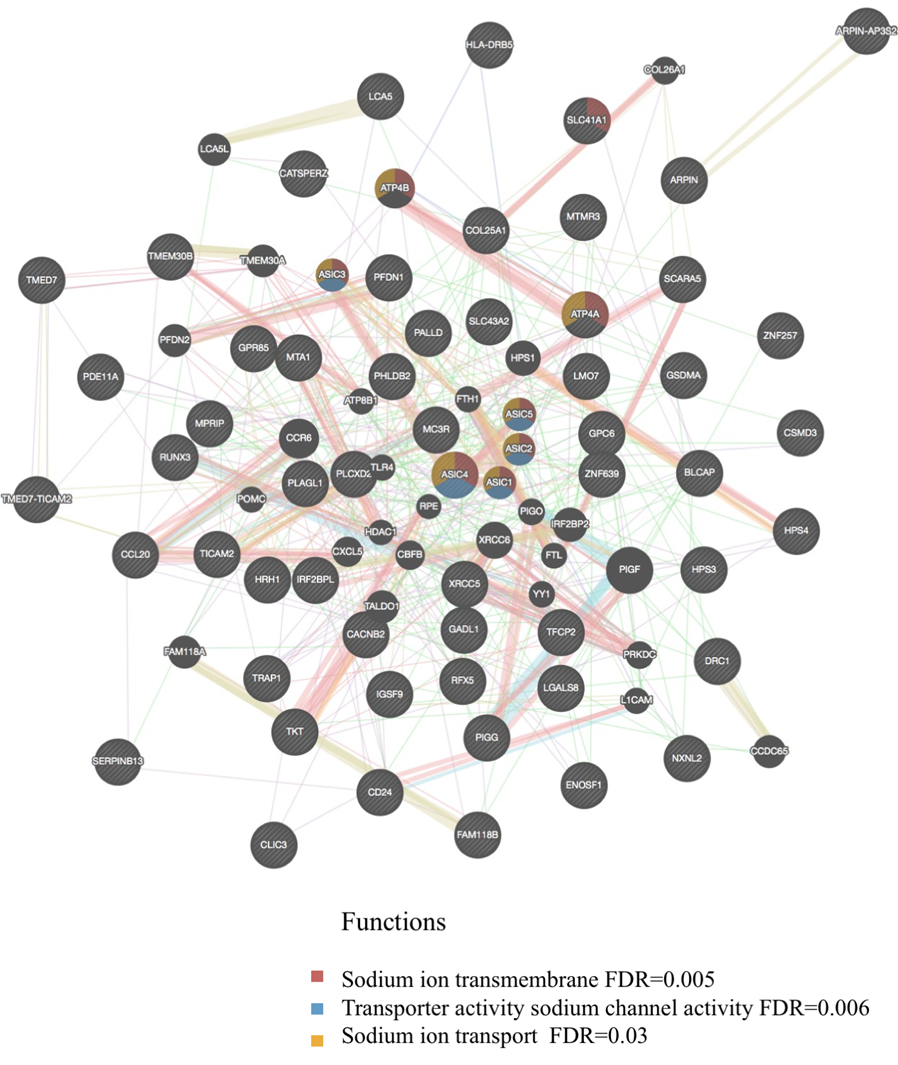


Figure S1: Network analysis for genes with hypomethylated DMPs identified for the vitamin D and high exercise group, extracted from GeneMania. Significant biological pathways (FDR < 0.05) associated with the input genes and related genes are described in the legend. The input genes are indicated with stripes.

Table S9: Top 8 vitamin D genes (r2 ≥ 0.2) associated with the study factors.

| Gene name | ***Number of CpGs*** | Factor | Correlation coefficient (r2) | P-value |
| --- | --- | --- | --- | --- |
| *RXRA* | 1 | Age | -0.22 | 0.00023 |
| *PNPLA1* | 1 | Physical activity-summer | 0.21 | 0.00059 |
|  | 1 | Physical activity-winter | 0.22 | 0.00042 |
| *SERINC2* | 1 | Alcohol (SD/week) | -0.22 | 0.00025 |
| *SOAT1* | 1 | Physical activity-summer | 0.20 | 0.00081 |
|  | 1 | Age | 0.20 | 0.00093 |
| *SMARCA4* | 1 | Age | -0.21 | 0.00080 |
| *PPARGC1B* | 3 | Age | -0.27  -0.20  0.20 | 0.000009  0.00098  0.00100 |
| *ACSL1* | 1 | Age | 0.20 | 0.00083 |
| *SMARCC1* | 1 | Age | -0.21 | 0.00080 |
